# Secondary data analysis of Adverse Drug Reactions reported by healthcare professionals and patients: interventions to manage ADRs of ACE inhibitors and ARBs

**DOI:** 10.1007/s00228-026-04088-6

**Published:** 2026-06-03

**Authors:** S. H. Spronk, J. T. H. Nielen, L. Heier, N. Jessurun, A. Kant, F. Karapinar -Çarkit

**Affiliations:** 1 Department of Clinical Pharmacy and Toxicology, Maastricht University Medical Center+, Maastricht, The Netherlands; 2 Department of Clinical Pharmacy, NUTRIM, Institute of Nutrition and Translational Research in Metabolism, Maastricht University, Maastricht, The Netherlands; 3 Department of Clinical Pharmacy, CARIM, Cardiovascular Research Institute Maastricht, Maastricht University, Maastricht, The Netherlands; 4 Interdisciplinary Centre for Palliative Medicine, Medical Faculty, University Hospital Duesseldorf, Heinrich-Heine-University Duesseldorf, Duesseldorf, Germany; 5 The Netherlands Pharmacovigilance Centre Lareb, Goudsbloemvallei 7, ‘s-Hertogenbosch, The Netherlands; 6 Department of Clinical Pharmacy and Toxicology, Leiden University Medical Centre, Leiden, The Netherlands

**Keywords:** Prescribing cascades, Adverse drug reactions, Secondary data analysis, Cardiovascular agents

## Abstract

**Purpose:**

Prescribing cascades occur when an adverse drug reaction (ADR) of a first medication is treated with a second medication, potentially leading to polypharmacy and harm. While various interventions are recommended to prevent harm, it is unknown which are implemented in daily practice. This study aims to assess the interventions implemented for ADRs of angiotensin-converting enzyme (ACE) inhibitors and angiotensin receptor blockers (ARB).

**Methods:**

This cross-sectional study analysed secondary data from spontaneous ADR reports submitted to the Netherlands Pharmacovigilance Center Lareb. Reports were included if the ADR was suspected to be caused by an ACE inhibitor or ARB and an intervention was documented. The primary outcome was the proportion of ADRs treated with (1) dose reduction, (2) discontinuation of the suspected medication, (3) discontinuation followed by medication to treat the ADR, (4) switching to alternative medication, or (5) addition of marker medication (prescribing cascade). The secondary outcome was the proportion of ADRs resolved at the time of the ADR report.

**Results:**

A total of 902 ADR reports were included. The most frequent intervention was discontinuing the suspected medication, followed by introducing medication to treat the ADR (42.5%), resulting in a large proportion of resolved ADRs (46.5%). However, 11.9% of reports included prescribing cascades, resolving the ADR in only 25.2% of reports.

**Conclusion:**

Using real-world pharmacovigilance data, this study offers valuable insights into the treatment of ADRs of ACE inhibitors and ARBs in daily practice. Further research is needed to establish the most effective interventions for specific types of ADRs.

**Supplementary Information:**

The online version contains supplementary material available at 10.1007/s00228-026-04088-6.

## Introduction

Prescribing cascades occur when an adverse drug reaction (ADR) of a first medication (the index medication) is treated with a second medication (the marker medication) [1]. Prescribing cascades can be intentional and appropriate, for example to prevent a specific ADR, such as adding a laxative when prescribing an opioid to prevent obstipation. However, prescribing cascades can also occur unintentionally, for example when an ADR is not recognized as such and treated as a new condition. Such prescribing cascades are defined as inappropriate prescribing cascades, since they result in unnecessary medication use and possibly contribute to polypharmacy and additional ADRs [1–4]. Furthermore, such cascades can cause harmful effects, can have a considerable impact on a patient’s health, and therefore result in increased costs due to additional risks and treatment. For example, a study by Rochon et al. (2024) showed that patients experiencing a prescribing cascade (i.e., a calcium channel blocker followed by a diuretic) had a higher risk of serious adverse events, including emergency room visits or hospitalizations [5, 6].

Despite increasing awareness in the last years, it remains difficult for healthcare professionals (HCPs) to manage prescribing cascades [5]. This may be primarily because prescribing cascades often occur in older patients with multiple morbidities, polypharmacy, and multiple HCPs involved in a patient’s care. HCPs may not have access to relevant information or a complete overview of the patient’s medication, making it more challenging to manage ADRs [7–12].

Timely management of an ADR can help prevent prescribing cascades. A systematic review by Adrien et al. (2023) provides an overview of 115 prescribing cascades, including recommendations for 52 prescribing cascades for HCPs to reverse or prevent them. The recommendations included switching to alternative medication, discontinuation of the index medication, or a dose reduction of the index medication for ADRs that are dose dependent [7]. Treating the ADR with a marker medication should be a last resort option, as there are generally better alternatives, and a marker medication does not always adequately treat the ADR [3, 7, 13]. However, it is unknown what interventions are conducted in daily practice to manage ADRs, and whether these interventions effectively resolve the ADR.

Therefore, the primary aim of this study was to assess the proportion of different types of interventions reported to manage ADRs of angiotensin-converting enzyme (ACE) inhibitors and angiotensin receptor blockers (ARBs). The study focused on ADRs of ACE inhibitors and ARBs, since these agents are known to be involved in multiple prescribing cascades [4, 7, 14, 15]. Furthermore, the proportion of ADRs that was resolved at the time of the ADR report after the different types of interventions was evaluated.

## Methods

### Study design and data setting

This was a descriptive, cross-sectional study using secondary data analysis. For the description of the study design, data collection and data analysis, the STROSA guidelines (Standardized Reporting Of Secondary data Analysis) were used (Supplementary Material 1) [16]. This study was approved by the Ethics Committee of Maastricht University Medical Centre (MUMC+) and Maastricht University on November 27th, 2024 (METC 2024 − 0487).

The data were extracted from spontaneously reported ADRs that were submitted to the Netherlands Pharmacovigilance Center Lareb (Lareb) between 26 June 2019 and 2 January 2025. A research agreement was signed between Lareb and Maastricht University for conducting secondary data analysis. Lareb is the Dutch national reporting and knowledge center for ADRs. Lareb receives reports of suspected ADRs through spontaneous reporting from HCPs and patients that could provide valuable insights in the occurrence of prescribing cascades and the intervention options chosen [17]. Reports can be submitted at any point in time after an ADR has occurred, and everyone is allowed to report an ADR. Patients can either report their own experiences, or a relative or HCP can submit a report. Additionally, multiple ADRs can be reported for an individual patient. Consequently, the data consisted of suspected ADRs, but were not evaluated for causality. ADRs are mostly reported digitally using a web-based reporting form, which can be assessed at www.lareb.nl [17]. The reporting form consists of, among others, questions regarding patient characteristics (e.g., age, sex, concomitant medications), the medication that is suspected of causing the ADR (e.g., name of the medication, indication, dose), and any potential interventions that were taken for the medication (e.g., dose reduction of the suspected medication, discontinuation of the suspected medication, dose not changed), as well as the outcome of the ADR (i.e., resolved, still resolving, not resolved, or fatal).

### Inclusion and exclusion of ADR reports

ADR reports were included in the dataset if the ADR was suspected to be caused by an ACE inhibitor or ARB. This selection was based on Anatomical Therapeutic Chemical (ATC) classification codes (C09A, C09B, C09BX, C09C, C09D). This also includes combination products (e.g., ARB + diuretic, ARB + calcium channel blocker). Furthermore, only ADR reports that documented an intervention were included. Duplicate reports, regarding the same ADR in the same patient, were excluded.

### Data collection and classification

Anonymized data was provided by Lareb. The variables that were collected included the type of reporter, sex and age of the patient experiencing the ADR, the suspected medication, the indication for the suspected medication, the type of ADR (classified using MedDRA terminology [18]), the outcome of the ADR, and the type of intervention that was reported. These variables could be reported in structured fields, and, in the case of interventions, also as free text. Classification of interventions was primarily based on structured data, specifically the responses to the question in the reporting form regarding the action taken with the suspected medication. This structured field had the following options: drug withdrawn, dose reduced, dose increased, dose not changed, or unknown. However, these categories do not provide information regarding the start of marker medication or a switch to alternative medication. For this reason, we additionally analysed unstructured data from the free text fields, adding information on how the ADR was treated. If it was mentioned that additional medication was started without discontinuation of the suspected medication (i.e., the action taken was dose reduced, dose increased or dose not changed), the intervention was classified as initiation of a marker medication. If it was mentioned that the patient switched to alternative medication, the intervention was classified as a switch. This does not include switching to a different brand of the same medication. All reports were assessed individually and the interventions were classified by two researchers. In cases of uncertainty or disagreement, the two researchers discussed the case until consensus was reached. In cases of missing data, the interventions were classified as unknown.

### Outcome measures

The primary outcome of this study was the proportion of different types of interventions reported to manage ADRs (1) treated with a dose reduction of the suspected medication, (2) treated with discontinuation of the suspected medication, (3) treated with discontinuation of the suspected medication followed by introduction of other medication to treat the ADR, (4) treated with a switch to alternative medication, or (5) treated with a marker medication (i.e., a prescribing cascade). The secondary outcome included the proportion of reported ADRs that were resolved at the time of the reporting of the ADR after the aforementioned interventions.

### Data analysis

Descriptive statistics were used to analyse patient characteristics, primary, and secondary outcomes. For patient characteristics, percentages were used to report categorical variables and mean values were used to report continuous variables. Percentages were used to report the proportion of ADRs treated with the different types of interventions and the outcome of the ADRs at the time of the ADR report after these interventions. Statistical analyses were performed using IBM SPSS Statistics 28.

## Results

In total, 911 ADRs suspected to be caused by an ACE inhibitor or ARB were collected. After excluding 9 duplicates, 902 ADR reports were included in the data analyses (Table 1). A variety of ADRs were reported, with the most commonly reported ADRs being angioedema (8.4%), headache (2.8%), and cough (2.4%). These ADRs were experienced by 487 different patients. The mean number of ADRs per patient was 1.85, with a range of 1 to 12. 55.6% of the patients were female, and the mean age was 62.8 years. The most frequently reported indication for the ACE inhibitor or ARB was a cardiovascular disorder (e.g., hypertension or heart failure). 64.1% of all ADRs were reported by patients themselves or their relatives, and 32.8% were reported by physicians or pharmacists, and the remainder by other HCPs (e.g., nurses).

**Table 1:** Patient characteristics

Patient characteristics (*n* = 487)	
Sex	
Male, n (%)	216 (44.4)
Female, n (%)	271 (55.6)
Age, mean (sd)	62.8 (14.1)
ADR report characteristics (*n* = 902)	
Type of cardiovascular agent^a^	
ACE inhibitor, n (%)	504 (55.9)
ARB, n (%)	398 (44.1)
Indication of cardiovascular agent	
Cardiovascular disorders, n (%)	783 (86.8)
Unknown indication, n (%)	42 (4.7)
Neurological disorders, n (%)	34 (3.8)
Multiple indications, n (%)	13 (1.4)
Kidney and renal disorders, n (%)	6 (0.7)
Other, n (%)	2 (0.2)
Missing, n (%)	22 (2.4)
Type of reporter	
Patient or their relative, n (%)	578 (64.1)
Physician, n (%)	229 (25.4)
Pharmacist, n (%)	67 (7.4)
Other healthcare professional (e.g., nurse), n (%)	28 (3.1)
Top 5 of ADRs	
Angioedema, n (%)	76 (8.4)
Headache, n (%)	25 (2.8)
Cough, n (%)	22 (2.4)
Dizziness, n (%)	16 (1.8)
Pruritus, n (%)	16 (1.8)
Other, n (%)	747 (82.8)

*ACE* angiotensin-converting enzyme, *ADR* adverse drug reaction, *ARB* angiotensin receptor blocker

^a^Two patients were using both an ACE inhibitor and an ARB

In 383 reports (42.5%), the suspected medication was discontinued, and other medication was started to treat the ADR (Table 2). In 186 reports (20.6%), the suspected medication was discontinued without additional treatment. Marker medication, and subsequently a prescribing cascade, was introduced in 107 reports (11.9%) to treat the ADR. Furthermore, in 75 reports, the suspected medication was switched to alternative medication (8.3%). The dose of the suspected medication was reduced in 39 reports (4.3%), and increased in 1.0% of reports. In 45 reports (5.0%), the suspected medication was not changed, and other types of interventions were initiated (e.g., physiotherapy, diagnostic monitoring). At the time of the reporting of the ADR, 359 ADRs had resolved (39.8%), 278 ADRs were still resolving (30.8%), and 185 ADRs were unresolved (20.5%)(Table 2). Notably, one of the reported ADRs, subglottic oedema, had a fatal outcome (0.1%).

**Table 2:** Frequency of intervention types and outcomes

Interventions, *n*	902
Discontinuation followed by introduction of other medication to treat the ADR, n (%)	383 (42.5)
Discontinuation, n (%)	186 (20.6)
Introduction of marker medication (i.e., a prescribing cascade), n (%)	107 (11.9)
Switch to alternative medication, n (%)	75 (8.3)
Intervention unknown, n (%)	58 (6.4)
No change in the suspected medication, n (%)	45 (5.0)
Dose reduction, n (%)	39 (4.3)
Dose escalation, n (%)	9 (1.0)
Outcomes^b^, n	902
ADR resolved, n (%)	359 (39.8)
ADR still resolving, n (%)	278 (30.8)
ADR unresolved, n (%)	185 (20.5)
Outcome unknown, n (%)	79 (8.8)
Fatal ADR, n (%)	1 (0.1)

*ADR*  adverse drug reaction

^b^At the moment of reporting

Discontinuation of the suspected medication was most often reported to resolve the ADRs (Table 3). At the moment of reporting, specifically, 50.0% of ADRs resolved (*n* = 93) after discontinuation of the suspected medication, and 46.5% of ADRs resolved after discontinuation of the suspected medication and introduction of other medication to treat the ADR (*n* = 178). Switching to alternative medication resolved 45.3% of ADRs (*n* = 34). Dose reduction of the suspected medication resulted in resolution of the ADR in 33.3% of reports (*n* = 13). The introduction of marker medication, which led to a prescribing cascade, resolved 25.2% of ADRs (*n* = 27). Following this type of intervention, the largest proportion of ADRs were unresolved. At the moment of reporting, 43.0% of ADRs remained unresolved (*n* = 46).

**Table 3:** Outcomes according to different intervention types

	ADRs resolved, *n* (%)	ADRs still resolving, *n* (%)	ADRs unresolved, *n* (%)	ADRs that were fatal, *n* (%)	Outcome unknown, *n* (%)
Discontinuation of the suspected medication followed by introduction of other medication to treat the ADR, *n* = 383	178 (46.5)	152 (39.7)	30 (7.8)	1 (0.3)	22 (5.7)
Discontinuation of the suspected medication, *n* = 186	93 (50.0)	43 (23.1)	30 (16.1)	0 (0)	20 (10.8)
Introduction of marker medication (i.e., a prescribing cascade), *n* = 107	27 (25.2)	24 (22.4)	46 (43.0)	0 (0)	10 (9.3)
Switch to alternative medication, *n* = 75	34 (45.3)	14 (18.7)	24 (32.0)	0 (0)	3 (4.0)
Intervention unknown, *n* = 58	5 (8.6)	8 (13.8)	25 (43.1)	0 (0)	20 (34.5)
No change in the suspected medication, *n* = 45	8 (17.8)	19 (42.2)	16 (35.6)	0 (0)	2 (4.4)
Dose reduction of the suspected medication, *n* = 39	13 (33.3)	11 (28.2)	13 (33.3)	0 (0)	2 (5.1)
Dose escalation of the suspected medication, *n* = 9	1 (11.1)	7 (77.8)	1 (11.1)	0 (0)	0 (0)

*ADR* adverse drug reaction

Table 4 provides an overview of how the most frequently reported ADRs were treated in daily clinical practice, and to what extent these interventions resolved the ADR at the moment of reporting. Angioedema, cough, and dizziness were mainly treated with discontinuation of the suspected medication, and in the case of angioedema, other medication was introduced. These interventions resolved 57.9%, 33.3%, and 50.0% of the ADRs, respectively. In contrast, headache and pruritus were mainly treated with marker medication, which resolved the ADRs in 38.5% and 0.0% of reports, respectively.

Last, the most frequently reported prescribing cascades included the following (not shown in Table 4): pruritus or related symptoms (e.g., rash) treated with topical corticosteroids or antihistamines; headache treated with analgesics such as paracetamol or ibuprofen, flu-like symptoms (e.g., fever, malaise) treated with analgesics, and cough treated with codeine or acetyl cysteine. The majority of prescribing cascades were reported by patients or their relatives (72%).

**Table 4:** Most frequent reported ADRs with associated interventions and outcomes

Type of ADR (*n*)	Reported interventions types for specific ADR, *n* (%)		Proportion of ADRs resolved after the intervention (%)
Angioedema (76)	Discontinuation and introduction of other medication to treat the ADR	67 (88.2)	57.9
	Discontinuation	2 (2.6)	50.0
	Introduction of marker medication	2 (2.6)	50.0
	Switch to alternative medication	3 (4.0)	66.7
	Intervention unknown	2 (2.6)	100
Headache (25)	Discontinuation and introduction of other medication to treat the ADR	5 (20.0)	20.0
	Discontinuation	2 (8.0)	100
	Introduction of marker medication	13 (52.0)	38.5
	Switch to alternative medication	2 (8.0)	0.0
	No change in the suspected medication	3 (12.0)	0.0
Cough (22)	Discontinuation and introduction of other medication to treat the ADR	7 (31.8)	28.6
	Discontinuation	9 (40.9)	33.3
	Switch to alternative medication	4 (18.2)	75.0
	No change in the suspected medication	1 (4.5)	0.0
	Dose reduction	1 (4.5)	0.0
Dizziness (16)	Discontinuation and introduction of other medication to treat the ADR	3 (18.8)	0.0
	Discontinuation	4 (25.0)	50.0
	Switch to alternative medication	2 (12.5)	50.0
	Intervention unknown	2 (12.5)	0.0
	No change in the suspected medication	3 (18.8)	0.0
	Dose reduction	1 (6.3)	100.0
	Dose escalation	1 (6.3)	0.0
Pruritus (16)	Discontinuation and introduction of other medication to treat the ADR	6 (37.5)	50.0
	Discontinuation	3 (18.8)	66.7
	Introduction of marker medication	6 (37.5)	0.0
	Intervention unknown	1 (6.3)	0.0

## Discussion

### Summary of main findings

Timely management of ADRs is essential to prevent and reverse inappropriate prescribing cascades. Secondary data analyses show that, if an ADR suspected to be caused by an ACE-inhibitors or ARB is reported, the most frequently chosen intervention by physicians in the Netherlands was the discontinuation of the suspected medication (20.6%), in the majority of cases followed by the introduction of other medication to treat the ADR (42.5%). The addition of new medication to treat the ADRs is explainable given the burden of the ADR and risk of a severe progression of certain ADRs if left untreated. For example, angioedema was the most frequently reported ADR and is usually treated with medications such as adrenaline, among others [19]. These two types of interventions also resulted in the largest proportions of resolved ADRs (50.0% and 46.5% respectively). This suggests that discontinuation of the suspected medication is the most effective strategy to treat ADRs.

However, discontinuation of the suspected medication may not always be desirable. Notably, fewer physicians chose to reduce the dose of the suspected medication (4.3%). This resulted in resolution of the ADR in 33.3% of cases at the time of reporting. Therefore, this intervention may be valuable in cases where discontinuation of the suspected medication initially is not considered an option. Nevertheless, dose reduction will only be effective if the ADR is dose-related.

Despite the fact that prescribing cascades should be a last resort option, they were introduced in 11.9% of cases. This seemed to be the least effective strategy, resolving the ADR in only 25.2% of cases at the time of reporting. Therefore, this study suggests that other interventions may be more effective in managing ADRs than introducing marker medications, which aligns with the recommendations of the systematic review of Adrien et al. (2023) [7]. However, more information is needed to manage ADRs, as this systematic review for example showed that many studies failed to specify how to manage ADRs resulting in prescribing cascades. Furthermore, the majority of prescribing cascades were reported by patients and also included over-the-counter medication. The reports did not capture whether this medication was purchased by the patient himself or prescribed or advised to the patient by a HCP. In addition, it is important to consider the possibility that HCPs may not always have been informed by patients about the ADRs they were experiencing. Consequently, patients may have used additional medication to manage their ADR without consulting their HCP.

The results of this study are consistent with those reported by Srisuriyachanchai et al. (2023), who found that ADRs were most frequently managed by physicians with drug withdrawal in 84.7% of cases [20]. However, this percentage is higher than the percentage observed in the present study, which may be due to differences in most frequent reported ADRs and suspected medications. This study focuses on ACE-inhibitors and ARBs, whereas Srisuriyachanchai et al. (2023) also included a considerable number of antibiotic-induced skin tissue disorders, which potentially require other types of interventions. In addition, in the study of Srisuriyachanchai et al. (2023), physicians also chose to introduce other medications to treat the ADR in 28.5% of cases. They did not assess whether this resulted in prescribing cascades.

### Strengths and limitations

The main strength of this study is that it is the first study to use nationwide reports to explore how ADRs of ACE inhibitors and ARBs are managed and whether prescribing cascades occur. By using real-world pharmacovigilance data, this study offers a valuable insight into the decisions physicians make in daily practice. Furthermore, in addition to structured data, free text fields were analysed. This provided more detailed information on the interventions that were taken and offered additional insights that otherwise might have been missed (e.g., the development of prescribing cascades). This contributed to the broad range of intervention types included in the analyses. Last, by including ADR reports from both patients and HCPs, this study ensured that patients’ perspectives were represented in addition to clinical viewpoints of HCPs.

However, this study also has some limitations. First, ADR reports could be submitted at any point in time after the occurrence of the ADR. As a consequence, some ADRs were still resolving at the time they were reported. Because of missing longitudinal data, it remains unclear whether these ADRs eventually resolved, indicating that the intervention was effective, or if they persisted. This may have led to an underestimation of the number of resolved ADRs. It is also possible that other interventions or prescribing cascades were initiated after the time of the report, which were missed in this study.

Another aspect that should be considered is when interpreting the results, is the reliability and completeness of the information reported. The information provided in individual reports was variable and did not include detailed information about clinical reasoning, shared decision making, or medication changes. Moreover, there was no information available regarding the severity of the reported ADRs. As a result, it remains unclear how the severity of an ADR influences the intervention chosen.

Additionally, this study did not evaluate differences in outcomes between various types of ADRs or specific ACE inhibitors or ARBs. It is possible that not all types of ADRs respond equally to the same intervention. For example, dose-related ADRs may be effectively treated by a dose reduction of the suspected medication, whereas ADRs that are not dose-related may not respond to this intervention.

Furthermore, the dataset consists of spontaneously reported ADRs only, which suggests a potential selection bias and may not accurately represent all ADRs, limiting the generalizability of our results. Selection bias may also have been introduced due to the inclusion of only ADRs for which treatment was reported. However, it remains unclear whether all interventions for ADRs are reported. Additionally, regarding the different types of interventions, the report does not specifically ask whether patients switched to alternative medication. Some reporters mention this voluntarily, but it is unclear if this is consistently reported. These limitations in reporting may have resulted in an underestimation of certain interventions. Also the incidence of interventions and prescribing cascades could not be calculated, since a spontaneous reporting system was used [21].

Last, in probably almost all reports there was at least a suspicion of an ADR, but causality assessment was lacking. Therefore, prescribing cascades due to unrecognized ADRs were not captured in this study. In case of combination products, the specific active ingredient responsible for the ADR was not assessed. This may lead to overestimation of the number of ADRs, as the ADRs could also be caused by the secondary medication of the combination product. Since it was difficult to determine which active ingredient caused the ADR, it was not possible to make a clear distinction. Therefore, all ADR reports were included for the sake of completeness.

## Conclusion

By using real-world pharmacovigilance data, this study offers a valuable insight into the management of ADRs, caused by ACE inhibitors and ARBs, in daily practice. Discontinuation of the suspected medication is the most commonly chosen intervention for the management of ADRs of ACE inhibitors and ARBs in the Netherlands. However, a considerable number of physicians also chose to introduce marker medication to treat the ADR, which led to a prescribing cascade. Therefore, it is important for physicians to focus more on alternative interventions in order to prevent inappropriate prescribing cascades and to effectively treat ADRs of ACE inhibitors and ARBs. However, future research is necessary to establish the most effective interventions for specific types of ADRs and to monitor if ADRs resolve over time.

## Supplementary Information

Below is the link to the electronic supplementary material.

Supplementary Material 1 (PDF 518 KB)

## Data Availability

The data that support the findings of this study are not publicly available, due to the data protection policy of the Netherlands Pharmacovigilance Center Lareb. Requests to access the dataset should be directed to Lareb. Representatives from Lareb were involved in all stages of this study (planning, interpretation, and implementation).
