# Supplementary material for: Secondary data analysis of Adverse Drug Reactions reported by healthcare professionals and patients: interventions to manage ADRs of ACE inhibitors and ARBs
Source: Eur J Clin Pharmacol. 2026 Jun 3;82(6):162. doi: 10.1007/s00228-026-04088-6 (PMC13234003; doi:10.1007/s00228-026-04088-6)
Supplement: Supplementary file 1 — Supplementary Material 1 (PDF 518 KB) [file 228_2026_4088_MOESM1_ESM.pdf]

European Journal of Clinical Pharmacology

Secondary data analysis of Adverse Drug Reactions reported by healthcare professionals and patients: interventions to manage ADRs of ACE inhibitors and ARBs

Spronk, S.H. [1,2], Nielen, J.T.H. [1,3], Heier, L. [4], Jessurun, N.[5], Kant, A. [5,6], Karapinar - Çarkit, F. [1,3]

[1] Department of Clinical Pharmacy and Toxicology, Maastricht University Medical Center+, Maastricht, The Netherlands
[2] Department of Clinical Pharmacy, NUTRIM, Institute of Nutrition and Translational Research in Metabolism, Maastricht University, Maastricht, The Netherlands
[3] Department of Clinical Pharmacy, CARIM, Cardiovascular Research Institute Maastricht, Maastricht University, Maastricht, The Netherlands
[4] Interdisciplinary Centre for Palliative Medicine, Medical Faculty and University Hospital Duesseldorf, Heinrich-Heine-University Duesseldorf, Duesseldorf, Germany
[5] The Netherlands Pharmacovigilance Centre Lareb, Goudsbloemvallei 7, 's-Hertogenbosch, The Netherlands
[6] Department of Clinical Pharmacy and Toxicology, Leiden University Medical Centre, Leiden, The Netherlands

Corresponding author: Karapinar - Çarkit, F. (f.karapinar@mumc.nl)

Supplementary Material

Supplementary 1: Standardized Reporting Of Secondary data Analysis (STROSA) checklist

	No.	Recommendation	Page no.
**Title and abstract**	1	a) Indicate in the title and abstract that the study is based on secondary data and name the origin of the data.<br>b) Write a structured summary stating the most important characteristics of the study design including study population and case definition. List secondary data-specific strengths and weaknesses of the study.	1<br>1
**Introduction**			
Background and rationale	2	Explain the scientific background and rationale for the study presented. Justify the choice of access via secondary data. Name the target group and the context of use of your study.	1-2
Objectives	3	All hypotheses and objectives. Due to the often data-driven approach to secondary data analyses, you can clearly identify whether the question is exploratory or hypothesis-testing.	2
**Methods**			
Study design	4	Clarify that the study is based on secondary data, including the primary purpose of the study and whether you have chosen a cross-sectional, cohort, case-control or other study design within your secondary data analysis.	2
Frame	5	Name the data source, its origin, the original purpose, the data owner, the reference population and the time period of the collected data.	2
**Legal basis**	6	Please describe the contractual and data protection law basis on which the Data provided and subsequently analyzed.	2
**Data flow**	7	The data flow indicate by whom the data is provided and where the data analyzed.	2-3
**Curriculum**	8	Explain whether your analyses followed a predetermined study protocol and to what extent your study has an exploratory and/or hypothesis-testing character.	2-3
**Analyses unit**	9	Define your units of analysis clarify whether the data represents a case and/or have a reference to persons or insured persons.	2-3
Study participants	10	Describe whether within the secondary data corpus a complete survey or a sampling has taken place. Describe inclusion and exclusion criteria for the inclusion of study participants and, if applicable, matching criteria when using comparison groups.	2
**Internal validations**	11	Describe and justify the internal validation measures taken. (Diagnostic) validation.	2-3
Variables	12	Clearly define all target variables, exposures, predictors, possible Confounders and effect modifiers. Explain which of the fields contained in the secondary data were used and which new variables derived from them as part of the operationalization. Explain the socio-demographic data available in the secondary data.	2-3
Classification systems	13	Describe which classification systems are used for the documentation of diseases, impairments, medical services, etc. were used. Explain whether and, if so, how the information was validated.	2-3
Bias	14	Discuss the likelihood of selection bias and information bias and the measures you have taken to determine its presence and extent.	6
Study size	15	Explain how the study size was determined. Take into account information on clinical relevance (minimal clinically relevant difference) or relevance for the healthcare system. Justify the use of the entire available body of data or a randomized analysis.	2-3
Quantitative methods	16	Describe how you deal with counting events such as physician	2-3

		contacts, admissions, number of diagnoses etc. have been dealt with. If necessary describe how categories (groupings) were formed and why.	
Statistical methods	17	Describe all statistical methods, including the methods used for the control of confounding and for the description of subgroups and interactions. Explain how any missing data was handled. Describe any sensitivity analyses performed.	3
**Results**			
Selection of the study population	18	Visualize the process of selection from the original population to the study population, for example in the form of a flow chart.	3
Description of the participants	19	Describe the characteristics of the study participants (e.g. demographic, clinical and social characteristics) as well as exposures and possible confounders. Take into account whether there is a case or personal reference.	3
Statistical measures	20	Report the number of target events or statistical measures (e.g. mean and standard deviation) or number of participants in each exposure category.	3-4
Main results	21	Provide standardized and/or adjusted measures including raw scores and their precision. If necessary, consider providing estimates of relative and/or absolute risks for meaningful time periods.	4
Further analyses	22	If applicable, report on further analyses carried out, e.g. the analysis of subgroups and interactions as well as sensitivity analyses.	4
**Discussion**			
Main results	23	Summaries the most important results with regard to the study objectives. Before categorizing your results, emphasize the secondary data character of your study.	4-5
Restrictions	24	Comment here on the validity of the secondary data with regard to its suitability for Answer your research question. Also address information that was not available due to the primary purpose of your secondary data source (e.g. confounding, validity of individual characteristics, consequences of case definition, exposure and outcome). Discuss the direction and extent of each possible bias.	5-6
**Strengths**	25	Weigh up potentials and limitations appropriately and identify the strengths and limitations of your organization of the secondary data analysis in relation to your study. Comment on possible alternative study approaches.	6
Interpretation	26	Make a cautious overarching interpretation of the results and consider the aims and limitations of the study, the results of other studies and other relevant evidence.	6
Transferability	27	Discuss the transferability (external validity) of the study results to other study populations.	6
**Further information** Financing	28	Indicate how the present study was financed and explain the Role of donors in planning, conducting, analyzing and interpreting the study.	7
**Role of data owner**	29	Explain the role of the data owner in planning, implementing, analyzing and interpretation of the study and under whose responsibility it published.	7

19
